# Differences in sensitivity to new therapies between primary and metastatic breast cancer: A need to stratify the tumor response?

**DOI:** 10.1002/cam4.5236

**Published:** 2022-09-13

**Authors:** Hubert Beaumont, Nathalie Faye, Antoine Iannessi, Emmanuel Chamorey, Catherine Klifa, Chih‐Yi Hsieh

**Affiliations:** ^1^ Median Technologies, Les Deux Arcs Valbonne France; ^2^ Centre Antoine Lacassagne Nice France; ^3^ ASLAN Pharmaceuticals Singapore Singapore

**Keywords:** biomarkers, Breast Neoplasms, Multidetector Computed Tomography, Tumor burden

## Abstract

**Objective:**

We compared therapeutic response of Varlitinib + Capecitabine (VC) versus Lapatinib + Capecitabine (LC) in patients with human epidermal growth factor receptor 2‐positive metastatic breast cancer after trastuzumab therapy by assessing changes in target lesion (TL) diameter and volume per location.

**Methods:**

We retrospectively analyzed the CT data of the ASLAN001‐003 study (NCT02338245). We analyzed TL size and number at each location focusing on therapeutic response from baseline to Week 12. We used TL diameter and volume to conduct an inter‐arm comparison of the response according to: RECIST 1.1; stratified per TL location and considering TLs independently. Multiple pairwise intra‐arm comparisons of therapeutic responses were performed. Considering TL independently, weighted models were designed by adding weighted mean TL responses grouped by location.

**Results:**

We evaluated 42 patients (88 TL) and 35 patients (74 TL), respectively, at baseline and Week 12.

We found reductions in breast TL burden in the VC arm compared to the LC arm (*p* = 0.002 (diameter), *p* < 0.001 (volume)). Responses and TL sizes at baseline were not correlated.

Explained variabilities of volume change per TL location, patient and patient:TL interaction were 36%, 10% and 4% (VC), and 13%, 1% and 23%, (LC).

A test of inter‐arm difference of responses yielded *p* = 0.07 (diameter), and *p* < 0.001 (volume).

**Conclusions:**

The therapeutic responses differed across tumors' locations; the magnitude of the differences of responses across the tumors' locations were drug‐dependent. Stratified analysis of the response by tumor location improved drug comparisons and is a powerful tool to understand TL heterogeneity.

## Graphical abstract

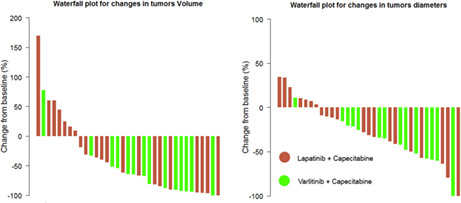

## 1 BACKGROUND

The Response Evaluation Criteria in Solid Tumors (RECIST) remain the most widely used criteria for assessing drug efficacy using imaging,^1^ primarily due to its simplicity and the lack of better established criteria.^2^ The heterogenous treatment responses observed in radiology following cytotoxic chemotherapy has already been reported.^3^ Now, some groups^4^, ^5^ have raised concerns about RECIST that may be suboptimal for assessing treatment response to new generations of therapeutics like tyrosine kinase inhibitors (TKIs), whose mechanisms of action (MoAs) differ from that of chemotherapy.

Since 2010, the Food and Drug Administration (FDA) started considering new types of anti‐cancer therapies^6^, ^7^ for which the pattern of response was neither observed nor considered when RECIST were developed. Since then, radiology has not evolved at the same pace as these new treatments have emerged.

Molecular intra‐tumor heterogeneity is often encountered with the use of TKIs, which rely on a novel MoA^8^, ^9^ and categorizing a disease as stable is often evidence of drug effectiveness.^10^, ^11^

Consequently, with new generations of anti‐cancer treatments, patterns of radiology response may vary with tumor locations^12^, ^13^ suggesting that sometimes, use of a stratified analysis would be more appropriate than a global one. Continued use of chemotherapy‐based response criteria for assessing clinical efficacy of new therapies is therefore suboptimal.^14^ Similar limitations apply when assessing clinical efficacy of cocktails of drugs or response in basket trials.

In recent years, there have been rapid developments in the field of quantitative imaging in radiology, with the release of guidelines for qualification of quantitative imaging biomarkers (QIBs)^15^ and recommendations for their implementation.^16^ Tumor volume in Computed Tomography (CT) has recently been presented as a valuable QIB that maxed all qualification steps.^17^

Coupling tumor volume as an advanced QIB with a stratified analysis of the therapeutic response per disease location may offer useful insight into drug efficacy. In our study, we compared therapeutic response of Varlitinib + Capecitabine (VC) versus Lapatinib + Capecitabine (LC) in patients with human epidermal growth factor receptor 2 (HER2)‐positive metastatic breast cancer (MBC) after trastuzumab therapy, using changes in tumor diameter and volume per tumor location.

## 2 MATERIALS AND METHODS

Our study was exempted by the Institutional Review Board (IRB) due to its retrospective nature. Written informed consent was not required as patient management was not impacted.

### 2.1 Data collection

We retrospectively analyzed CT scans measurements and annotations of 42 patients from the phase 2A multicenter ASLAN001‐003 clinical trial (NCT02338245), which compared the therapeutic response of VC versus LC in patients with HER2‐positive MBC after trastuzumab therapy. In the ASLAN001‐003 trial, RECIST 1.1 were applied and, additionally, changes in sum of target lesion (TL) volume were monitored. The ASLAN001‐003 trial used the LMS platform (Median Technologies, France) that automatically recorded tumor type, location, longest axial diameter (LAD), short axial diameter (SAD) and manually delineated volume.

Demographics and disease characteristics of the ASLAN001‐003 trial are summarized in Table 1. The key inclusion criteria were:

1. Documented histological confirmation of breast cancer with HER2 overexpression or gene amplification (immunohistochemistry 3+ or 2+ with fluorescent/chromogenic/silver in situ hybridization+) prior to study entry.
2. HER2 positive MBC that had progressed on prior first‐line treatment with trastuzumab in metastatic setting or relapsed within 1 year of treatment with trastuzumab in adjuvant setting.

**TABLE 1:** Demographic and disease characteristics of the ASLAN001‐003 clinical trial

Characteristic	Varlitinib + Capecitabine (*n* = 24)	Lapatinib + Capecitabine (*n* = 26)	All Patients (*N* = 50)
Age, median (range) y	53.5 (29–83)	56.5 (33–79)	55.0 (29–83)
Female sex, No. (%)	24 (100)	26 (100)	50 (100)
Ethnic origin, No (%)			
Asian‐Chinese	18 (75.0)	18 (69.2)	36 (72.0)
Asian‐other	3 (12.5)	4 (15.4)	7 (14.0)
White	2 (8.3)	2 (7.7)	4 (8.0)
Other	1 (4.2)	2 (7.7)	3 (6.0)
ECOG performance status, No. (%)			
0	19 (79.2)	20 (76.9)	39 (78.0)
1	3 (12.5)	6 (23.1)	9 (18.0)
2	2 (8.3)	0	2 (4.0)
Breast cancer status, No. (%)			
Recurrence	3 (12.5)	1 (4.8)	4 (8.0)
Metastasis	21 (87.5)	25 (96.2)	46 (92.0)
HER2 IHC, No. (%)			
1+	1 (4.2)	0	1 (2.0)
2+	6 (25.0)	9 (34.6)	15 (30.0)
3+	14 (58.3)	17 (65.4)	31 (62.0)
Missing	3 (12.5)	0	3 (6.0)
HER2 FISH, No. (%)			
Positive	10 (41.7)	12 (46.2)	22 (44.0)
Not performed	14 (58.3)	14 (53.8)	28 (56.0)

Abbreviations: ECOG, Eastern Cooperative Oncology Group; FISH, Fluorescence in situ hybridization; HER2, Human epidermal growth factor receptor 2; IHC, Immunohistochemistry; *N*, Number of patients in the trial; *n*, Number of patients in the treatment arms; No., Number; y, Years.

The key exclusion criteria were:

1. Patients who have received more than 2 lines of any therapies in metastatic stage, radiation treatment or major surgical procedures within 21 days prior to study entry.
2. Patients with any history of other malignancy unless in remission for more than 1 year.
3. Patients with an uncontrolled intercurrent illness.

### 2.2 Study workflow and analysis

For our study, measurements and annotations were automatically retrieved from the original trial database. Measurements (LAD, SAD and volume) and annotations recorded at baseline and Week 12 were quality controlled and analyzed by a 15Y+ medical imaging expert. Lymph nodes (LN) measurements (SAD) were specifically controlled to comply RECIST recommendations.

Our study plan was as follows:

#### 2.2.1 Population statistics

We compared the tumor size and the number of tumors at each disease location, and for each treatment arm.

#### 2.2.2 Inter‐arm comparison of the responses (Figure 1)

**FIGURE 1:**
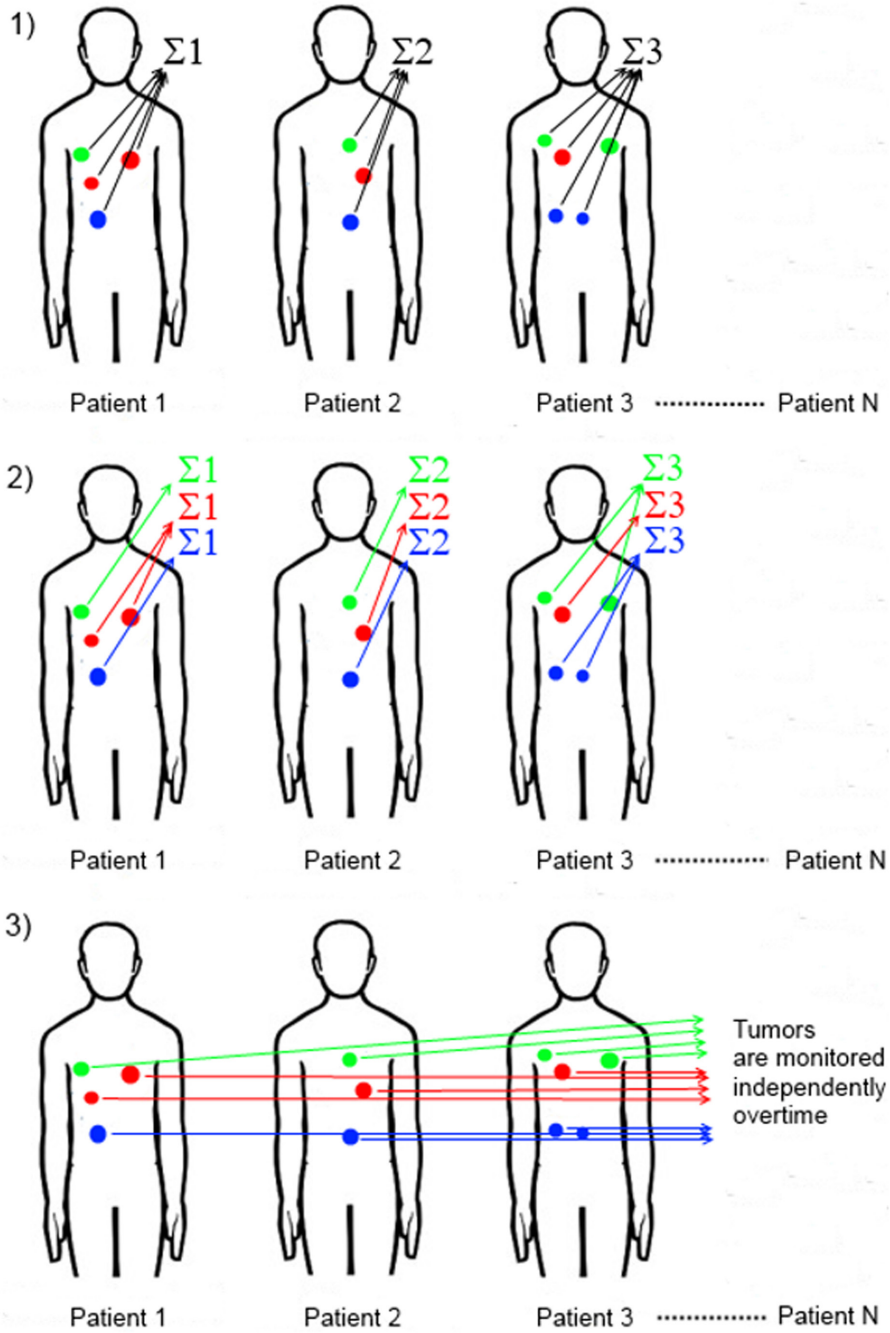
Inter‐arm comparisons of tumor responses. Three different analyses were performed. (1) Patient tumor burden was monitored according to RECIST 1.1. At each time point, for a given patient, the size of all target tumors was summed, and these sums were monitored over time. (2) Patient tumor burden was stratified by tumor location. At each time point, for a given patient, the size of target tumors from the same location was summed, and these sums were monitored over time. (3) All tumors were considered as independent from patients and monitored independently over time; the mean tumor change was computed per location.

We analyzed the mean changes of the tumor burden (as %) in considering:

1. The definition of tumor burden given by RECIST 1.1, where, for each patient, at each time point, the size (LAD/volume) of all TLs (up to 5 in number, independent of location, but no more than 2 per location) was summed, and these sums were monitored from baseline to Week 12;
2. Stratified tumor burden, where, for each patient, at each time point, the size (LAD/volume) of TLs from the same location were summed, and these sums were monitored from baseline to Week 12.
3. All tumors considered independent from patients and monitored from baseline to Week 12, with the average of tumor change in size (LAD/volume) computed per tumor location.

We tested if a significant relationship existed between tumor size at baseline and change from baseline at Week 12.

#### 2.2.3 Intra‐arm comparison of the responses

In each arm, (independently to patients) we grouped tumors by location then we compared the average response (as %) between these groups. We performed multiple pairwise comparisons of the responses between the various tumor locations (liver‐breast, lung‐breast, lymph node‐breast, lung‐liver, lymph node‐liver and lymph node‐lung). In using either LAD (SAD in the case of LN) or volume, we tested for significant differences at Week 12 in each treatment arm. Finally, we computed the mean tumor changes by stratifying patients' responses and did an analysis of variance (ANOVA).

#### 2.2.4 Modeling of the stratified response

Considering tumors independently from patients, we designed a model by adding mean tumor responses, grouped by locations and weighted by the proportion of tumors at these locations. The weighted model summarized the response to treatment. The model will be computed for each arm to allow for more accurate comparisons of inter‐arm responses. Treatment responses were summarized as follows:

$$\frac{1}{\sum \left({\mathrm{Nb}}_{i}\right)}*\sum \left({\mathrm{Nb}}_{i}*\bar{∆\left({\mathrm{TL}}_{i}\right)}\right)$$

With:

Nb_*i*_: Number of TLs at disease location *i* (*i* = breast, lung, liver or nodal tumors).

$\bar{∆\left({\mathrm{TL}}_{i}\right)}$: Mean change of TLs size (LAD or volume) at disease location _i_.

#### 2.2.5 Sensitivity analysis

We tested the robustness of our results by slightly changing the study input as follows^18^:

1. Excluding patients exhibiting extreme treatment response at Week 12, then re‐testing our conclusions with/without outliers;
2. Adjusting for the imbalance in number of independent tumors and number of patients after stratifying per tumor location at Week 12;

### 2.3 Statistics

The multiple comparisons of tumor sizes per tumor location were tested using Tukey Honest Significant Differences. Comparisons of tumor proportions at each location relied on a two‐sided Chi‐square test. We computed waterfall plots of patients' response (summing all tumors for each patient), and in stratifying patients' response per tumor location, and Wilcoxon‐rank tested the equivalence of inter‐arm and stratified intra‐arm responses.

Tests of multiple comparisons of tumor response per tumor location were performed applying Tukey Honest Significant Differences.

We used a two‐sided Chi‐square test for evaluating inter‐arm difference of response derived from the weighted models.

Eta‐squared derived from the ANOVA reported the proportion of explained variabilities.

As prerequisite of performing ANOVA, data were tested for homoscedasticity using Levene's test and for Normality using Jarque‐Bera test. Both tests are available from the “lawstat” R package.

Data were considered as outliers when outside the 1.5 Inter Quartile Range.^19^

The R 3.5.1 Cran software was used for statistics, *p* < 0.05 was considered a significant difference.

## 3 RESULTS

### 3.1 Population statistics

At baseline, 42 patients displayed at least 1 TL. A set of 88 TLs was distributed per disease location as follows: lung (31% *n* = 27), breast (26% *n* = 23), liver (23%, *n* = 20), lymph nodes (17% *n* = 15) and miscellaneous (3%; *n* = 3). Miscellaneous locations (skin and mediastinal lesions) were excluded as they were under‐represented. Therefore, 85 TLs were classified into 4 major groups by location (Table 2). To be noted that 22 patients had no visible primary breast tumors on CT due to previous trastuzumab treatment or because their tumors were visible only on mammography.

**TABLE 2:** Proportion of tumors at each disease location at baseline

	Varlitinib + Capecitabine		Lapatinib + Capecitabine		*p* value^a^
	Number of Tumors	%	Number of Tumors	%	
Breast	12	35.3	11	21.6	0.16
Lung	7	20.6	20	39.2	0.07
Liver	9	26.5	11	21.6	0.6
Lymph node	6	16.6	9	17.6	1.0
Total	34	100	51	100	

^a^ *p* value corresponding to statistical significance of the inter‐arm difference of proportion of tumor numbers, calculated using a two‐sided Chi‐square test.

At Week 12, 35 patients remained in the study (14 and 21 patients in the VC and LC arms, respectively) (Table 3) and 74 tumors were measured.

**TABLE 3:** Tumor burden changes from baseline to Week 12

	VC (tumor diameter)	LC (tumor diameter)	VC (tumor volume)	LC (tumor volume)
Number of evaluable patients	14	21	14	21
Mean change in tumor burden^a^	−40.03%	−21.19%	−64.15%	−25.59%
*p* value^b^	0.086		0.13	

Abbreviations: LC, Lapatinib + Capecitabine; VC, Varlitinib + Capecitabine.

^a^ Tumor burden computed as per Response Evaluation Criteria in Solid Tumor, by summing the size of up to 5 target tumors independent of tumor location, considering not more than 2 tumors per location.

^b^ *p* value corresponding to statistical significance of inter‐arm difference in mean changes in tumor burden (Wilcoxon rank test).

**FIGURE 2:**
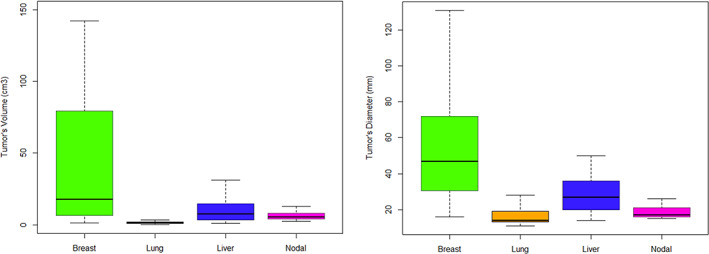
Boxplots showing distribution of tumor size at baseline per location of the disease (breast, lung, liver and nodal) with tumor volume (cm^3^) on the left and tumor diameter (mm) on the right. Breast tumors were larger, *p* < 0.001 for volume or diameter using Tukey Honest Significant Difference test.

Distributions of tumor size at baseline per tumor location in both treatment arms are displayed in Figure 2 for both QIBs.

At baseline, there was no significant difference between the treatment arms in the proportion of tumors (*p* = 0.27), though there was a greater proportion of lung tumors in the LC arm versus the VC arm (*p* = 0.07) (Table 2). When considering either QIB, the mean size of breast tumors was significantly larger than that of tumors at the other locations (*p* < 0.002).

### 3.2 Inter‐arm comparison of the responses

**FIGURE 3:**
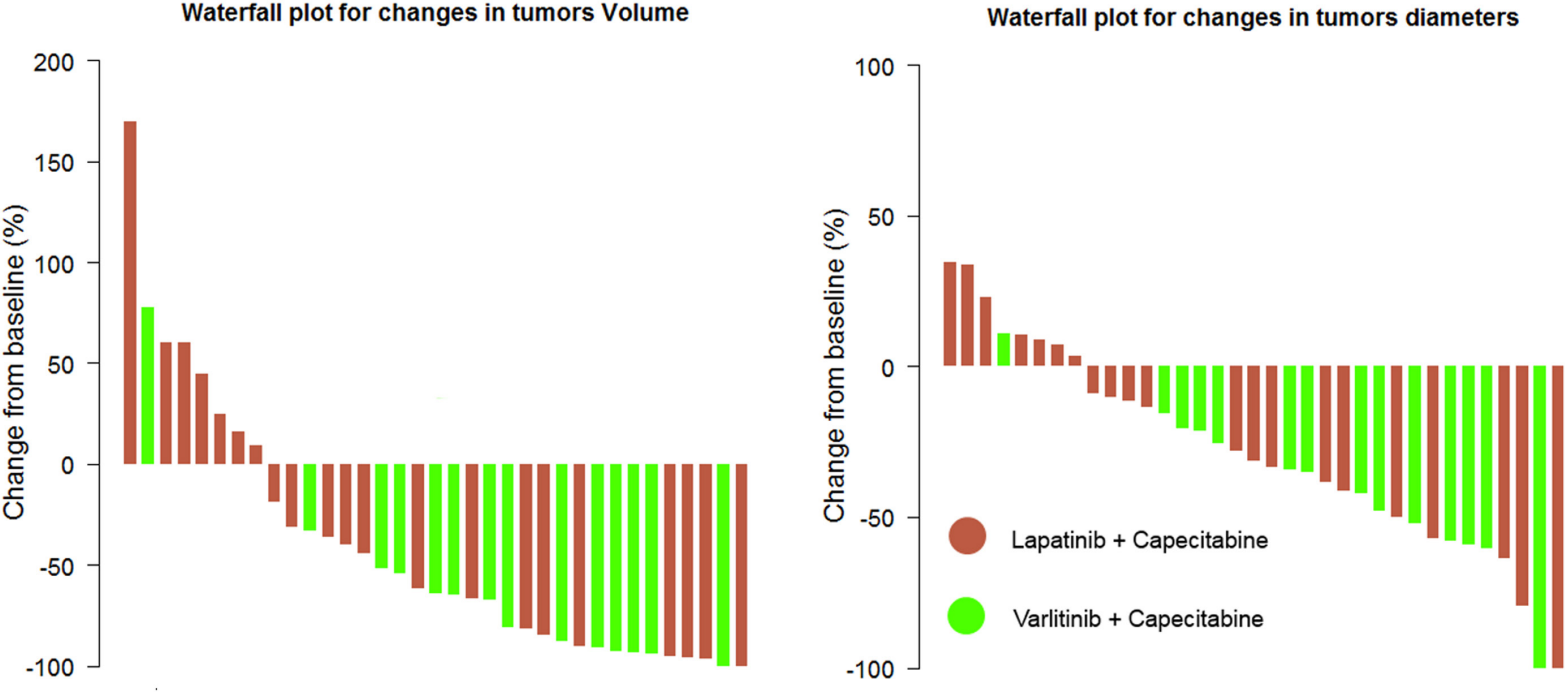
Waterfall plot showing changes from baseline at Week 12 in tumor volume (on left) and tumor diameter (on right). Green bars represent responses in the Varlitinib + Capecitabine arm; red bars represent responses in the Lapatinib + Capecitabine arm.

**FIGURE 4:**
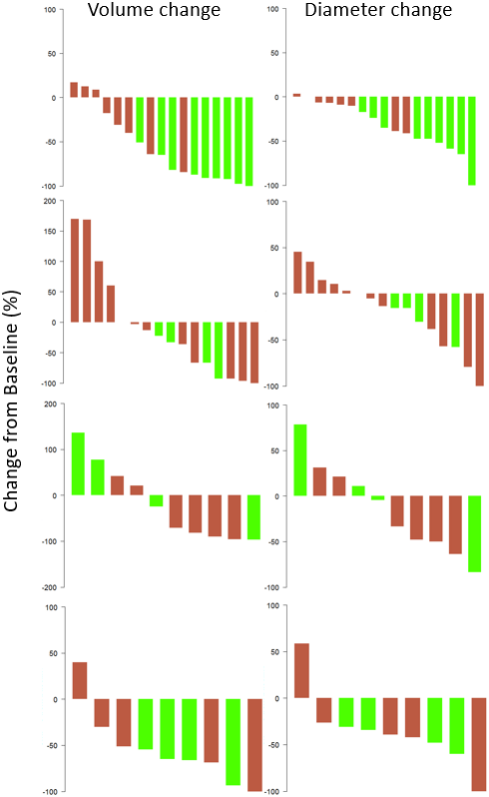
Waterfall plot showing stratified changes from baseline to Week 12 in tumor volume (on right) and diameter (on left) for breast, lung, liver, and lymph node tumors (from top to bottom). Green bars represent responses in the Varlitinib + Capecitabine arm; red bars represent responses in the Lapatinib + Capecitabine arm. There was significant inter‐arm difference only for changes in breast tumor burden (*p* = 0.002 and *p* < 0.001, respectively, for tumor diameter and volume as qualitative imaging biomarkers).

Tumor burden changes, in both treatment arms, are presented in Table 3. Waterfall plots of patient responses (LAD and volume) are displayed in Figures 3; changes of tumor burden stratified per tumor location are in Figures 4.

There were significant reductions in breast tumor burden in the VC arm compared to the LC arm (*p* = 0.002 for LAD, *p* < 0.001 for volume in favor of VC arm). No significant inter‐arm differences were noted for other TLs. Table 4 summarizes the mean tumor response with tumors considered independently from patients and grouped by location. Putting all tumors together without distinction from disease location and patient, a test of inter‐arm difference of the response yielded *p* = 0.02 for tumor LAD and *p* = 0.015 for tumor volume. There was no significant relationship between the response and baseline tumor size by LAD or volume.

**TABLE 4:** Mean proportional change (%) in diameter and volume of tumors considered independent of patients and grouped by disease locations

	Varlitinib + Capecitabine		Lapatinib + Capecitabine		*p* value^a^	
	Diameter (%)	Volume (%)	Diameter (%)	Volume (%)	Diameter	Volume
Breast	−50.19	−85.06	−16.15	−30.90	0.001	<0.001
Lung	−29.96	−56.31	−12.17	16.68	0.11	0.14
Liver	−8.64	7.24	−21.55	−44.71	0.80	0.40
Lymph node	−46.15	−71.7	−31.86	−46.48	0.82	0.59

^a^ *p* value corresponding to significance of the inter‐arm comparison of the proportional change according to diameter and volume of tumors (Wilcoxon rank test).

### 3.3 Intra‐arm comparison of the responses

Tables 5 and 6 summarize, for tumor diameter and volume, respectively, the difference of responses between the different pairs of tumor locations.

**TABLE 5:** Intra‐arm comparison of change in tumor diameter change at different tumor locations from baseline to Week 12

Mean proportional change in tumor diameter	Varlitinib + Capecitabine		Lapatinib+ Capecitabine	
	Difference [95% CIs] (%)	*p* value^a^	Difference [95% CIs] (%)	*p* value^a^
Liver‐breast	49.96 [−8.31; 108.29]	0.11	−10.08 [−70.42; 50.25]	0.97
Lung‐breast	19.89 [−38.41; 78.19]	0.77	−1.76 [−52.75; 49.23]	0.99
Lymph Node‐breast	6.33 [−51.96; 64.63]	0.99	−16.01 [−79.70; 47.67]	0.90
Lung‐liver	−30.09 [−98.69; 38.51]	0.61	8.32 [−47.53; 64.18]	0.98
Lymph node‐liver	−43.65 [−112.26; 24.95]	0.30	−5.93 [−73.58; 61.71]	0.99
Lymph node‐lung	−13.56 [−82.16; 55.03]	0.94	−14.25 [−73.72; 45.21]	0.91

Abbreviation: CI, Confidence interval.

^a^ *p* value corresponding to the test of a significant difference in the responses between the tumor locations (Test of Tukey Honest Significant Differences).

**TABLE 6:** Intra‐arm comparison of change in tumor volume at different tumor locations from baseline to Week 12

Mean proportional change in volume	Varlitinib + Capecitabine		Lapatinib+ Capecitabine	
	Difference [95% CIs] (%)	*p* value^a^	Difference [95% CIs] (%)	*p* value^a^
Liver‐breast	107.49 [26.34; 188.64]	0.007	−21.09 [−126.18; 83.98]	0.94
Lung‐breast	30.49 [−50.67; 111.64]	0.71	42.11 [−48.31; 132.52]	0.58
Lymph Node‐breast	14.47 [−66. 68; 95.62]	0.96	−17.16 [−128.08; 93.77]	0.97
Lung‐liver	−77.00 [−172.49; 18.49]	0.14	63.20 [−35.55; 161.96]	0.32
Lymph node‐liver	−93.02 [−188.51; 2.47]	0.057	3.94 [−113.88; 121.77]	0.99
Lymph Node‐lung	−16.01 [−111.51; 79.48]	0.96	−59.26 [−164.21; 45.69]	0.42

Abbreviation: CI, Confidence interval.

^a^ *p* value of the test of a significant difference in the responses between the tumor locations (Test of Tukey Honest Significant Differences).

For changes in tumor diameter (Table 5), explained variabilities per tumor location, patient and patient: tumor interaction were 22%, 5% and 16%, respectively, in the VC arm, and 2%, 0.5% and 30%, respectively, in the LC arm.

For changes in tumor volume (Table 6), explained variabilities per tumor location, patient and patient: tumor interaction were 36%, 10% and 4%, respectively, in the VC arm, and 13%, 1% and 23%, respectively, in the LC arm.

### 3.4 Model design

We applied our model using the distribution of tumor location (Table 2) and the average response by tumor location (independent tumors) in Tables 5 and 6. Thus, we modeled the response to treatment for the VC and LC arms, respectively, in Equations 1 and 2 (using LAD) and, respectively, in Equations 3 and 4 (using volume).

LAD

(1) $$\begin{array}{c} 1/28^{*}\;(11^{*}-50.19\%+5^{*}-29.96\%+6^{*}-8.64\%+6^{*}-46.15\%)=\\ -36.8\% \end{array}$$

(2) $$\begin{array}{c} 1/46^{*}\;\left(11^{*}-16.15\%+18^{*}-12.17\%+11^{*}-21.55\%+6^{*}-31.86\%\right)=\\ -17.9\% \end{array}$$

Volume

(3) $$\begin{array}{c} 1/28^{*}\;\left(11^{*}-85.06\%+5^{*}-56.31\%+6^{*}7.23\%+6^{*}-71.7\%\right)=\\ -57.3\% \end{array}$$

(4) $$\begin{array}{c} 1/46^{*}\;\left(11^{*}-30.90\%+18^{*}16.68\%+11^{*}-44.71\%+6^{*}-46.48\%\right)=\\ -17.6\% \end{array}$$

a test for a significant difference in inter‐arm responses yielded *p* = 0.07 (using LAD), and *p* < 0.001 (using volume) both in favor of VC arm.

### 3.5 Sensitivity analysis

The inter‐arm comparison of the stratified responses yielded *p* = 0.015 (using LAD) and *p* = 0.03 (using volume) after removing outliers at Week 12 (*n* = 6 for tumor diameter, *n* = 3 for tumor volume) (Table S1). When considering each tumor independently from patients, inter‐arm comparison yielded *p* < 0.007 (for tumor diameter) and *p* = 0.016 (for tumor volume) after removing outliers (*n* = 7 for tumor diameter, *n* = 4 for tumor volume). Intra‐arm comparisons of the stratified responses by disease location are summarized in Tables S2 and S3.

Equations 1.1–1.4 (electronic supplementary material [ESM]) obtained following data adjustment at Week 12 with balancing of tumors at each disease location were comparable to Equations (1), (2), (3), (4). A test for a significant difference in inter‐arm responses yielded *p* = 0.17 (tumor diameter) and *p* = 0.003 (tumor volume).

Equations 1.5–1.8 (electronic supplementary material [ESM]) obtained in computing stratified change of tumor burden, balancing numbers of patients having tumor at the same location, were comparable to Equations (1), (2), (3), (4). A test for a significant difference in inter‐arm responses yielded *p* = 0.26 for tumor diameter and *p* = 0.11 for tumor volume in favor of VC arm.

## 4 DISCUSSION

Our study showed that breast tumors were, on average, significantly larger than other tumors (*p* < 0.001). There was no significant inter‐arm difference in the proportion of tumors at different disease locations, though there was a greater proportion of lung tumors in the LC arm (*p* = 0.07). Inter‐arm tests showed a trend toward superiority of the VC arm per patient, and confirmed superiority of the VC arm when tumors were considered independently. Multiple intra‐arm comparisons showed that tumor volume is more sensitive than LAD for detection of differential responses at different disease locations. In the VC arm, we found a significant differential response between breast and liver tumors using volume (*p* = 0.007) and a trend toward superiority using volume in differential response for lymph node versus liver tumors (*p* = 0.057). No significant differences were measured in the LC arm using LAD or volume.

Results of the intra‐arm multiple comparisons confirmed the stratified inter‐arm results, showing a more favorable response in the VC arm compared to the LC arm, for both QIBs (*p* = 0.07 for LAD, *p* < 0.001 for volume). These results were also confirmed by the inter‐arm comparisons of the weighted models and the ANOVA, indicating a greater variability per tumor locations in the VC arm. The results of our study are strengthened by a sensitivity analysis that reported no significant impact of outliers upon our conclusions, and no change in the stratified responses of VC over LC, after adjusting the proportion of TLs at each disease location. Our stratified analysis showed the effectiveness of the drug at specific disease location. This insight would help to improve drug indications and to design more effective drug combinations.

Researchers have reported differential responses according to disease location. Menzies et al^20^ found significantly different Time To Best Response for subcutaneous soft tissue and lung metastases compared to lymph node and liver metastases, and Crusz et al^21^ found that 55.6% of patients showed a heterogeneous response. These studies drew contradictory conclusions regarding a relationship between tumor size at baseline and response. Our study did not show a relationship between tumor size at baseline and response. Usually, tumors have complex shapes and are heterogeneous; volumetric measurements have long showed better precision and accuracy than linear measurement, notably in advanced lung cancer patients.^17^, ^21^ However, very few studies have proved that changes in tumor volume better correlate to the disease or can be an alternative for clinical trial. In our study, we found that when tumor volumes were used, p values were lower when testing inter‐arm response according to a weighted model of stratified response *p* = 0.07 (for tumor diameter), and *p* < 0.001 (for tumor volume) or when tumors were all considered as independent *p* = 0.02 (for tumor diameter) and *p* = 0.015 (for tumor volume). We also found that tumor volume was more discriminant than diameter when testing differential response (e.g. *p* = 0.007 for liver‐breast in VC arm). Similar discrimination was not observed with RECIST (*p* = 0.13 for volume), (*p* = 0.086 for diameter). This can be explained by the design of RECIST that recommend adding tumors from different location, therefore losing the benefit of the volume.

We can also consider that the stratification of imaging therapeutic response represents a mean of investigation per say.^22^ It is known that spatial and temporal tumor heterogeneity can be due to the mutational status of tissues, their cellular morphology, metabolism, and proliferative and metastatic potential.^23^ Therapeutic response stratification can therefore be seen as an indirect noninvasive feedback on tumor heterogeneity. More specifically, the temporal monitoring of clinical data coupled with stratified responses could inform about different resistance mechanisms and their outbreak.^24^ Enriching biological data with stratified imaging responses would help to understand the MoA, identify drug sensitive or resistant cells and investigate new targeted therapy approaches.

Our study had some limitations, the first being that we analyzed tumor response over a short period of time. To match the ASLAN003‐001 trial setting, we restricted our analysis to Week 12. We may hypothesize that the stratified response at each tumor location can vary over time. For instance, at treatment onset, a drug could exhibit a superior efficacy upon primary breast tumors compared to metastases, which could fade, disappear, or even reverse over time. Because of the limited dataset we could not extend our study over multiple time points. A second limitation of our study was that we did not consider the aspect of measurement reliability. In our dataset, tumors had different size distributions according to locations, and the proportions of tumors at various locations differed slightly between the arms. Several groups have investigated the measurement reliability according to tumor size and location.^25^, ^26^ A more sophisticated model of stratified response would include the reliability of measurements as a parameter. A third limitation of our study was that it was not possible to consider all RECIST aspects as the unequivocal appearance of new lesions and progression of non‐target lesions (nTLs). In our study, at Week 12, a single new unequivocal new lesion was detected, 2 nTLs progressed while 9 decreased. The small data sizing precluded any significant conclusions.

A fourth limitation is inherent to the ASLAN003‐OO1 trial that mainly included Asian patients while Wagner et al.^27^ reminded that different response may exist between Asian and Caucasian ethnicities. Therefore, the generalizability of our observations needs to be confirmed with non‐Asian cohorts.

## 5 CONCLUSION

We found that drugs have different efficacy across tumor locations. In the era of new therapies, stratified analysis of response will provide better assessments and drug comparisons, and be a powerful tool contributing to improved understanding of the MoA behind tumor heterogeneity.

## AUTHOR CONTRIBUTIONS

Hubert Beaumont: Conceptualization, formal analysis, original draft, writing, review and editing. Nathalie Faye: Conceptualization, Data curation, review and editing. Antoine Iannessi: formal analysis, original draft, review and editing. Emmanuel Chamorey: methodology, formal analysis, review. Catherine Klifa: Conceptualization, methodology, project administration, review and editing. Chih‐Yi Hsieh: Data curation, review and editing. We certify that all co‐authors contributed equally and significantly to the study and to the design of the manuscript.

## FUNDING INFORMATION

This study did not received funding.

## CONFLICT OF INTEREST

The authors of this manuscript, Hubert Beaumont, Catherine Klifa, Nathalie Faye declare relationships with the following companies: Median Technologies. Chih‐Yi Hsieh declare relationships with ASLAN Pharmaceuticals. Other authors of this manuscript declare no relationships with any companies, whose products or services may be related to the subject matter of the article.

## ETHICS APPROVAL AND CONSENT TO PARTICIPATE

Our study was approved/waived by the Ethical Committee/Institutional Review Board (IRB) due to its retrospective nature.

Written informed consent was not required for this study because not impacting patient management.

## Supporting information


Table S1
Table S2
Table S3

## Data Availability

Study subjects or cohorts have been previously reported in ASLAN001‐003 clinical trial (NCT02338245).
