# Supplementary material for: Differences in sensitivity to new therapies between primary and metastatic breast cancer: A need to stratify the tumor response?
Source: Cancer Med. 2022 Sep 13;12(3):3112–22. doi: 10.1002/cam4.5236 (PMC9939226; doi:10.1002/cam4.5236)
Supplement: Supplementary file 1 — Table S1 Table S2 Table S3 [file CAM4-12-3112-s001.docx]

**ELECTRONIC SUPPLEMENTARY MATERIAL**

**Table I.1: Inter‑arm comparison of stratified responses from baseline to Week 12**

|  | **Varlitinib + Capecitabine** | | **Lapatinib+ Capecitabine** | | **p value**^a^ | |
| --- | --- | --- | --- | --- | --- | --- |
|  | **Tumor diameter (%)** | **Tumor volume (%)** | **Tumor diameter (%)** | **Tumor volume (%)** | **Tumor diameter (%)** | **Tumor volume (%)** |
| **Breast (N)** | -43.3 (8) | -84.2 (9) | -13.7 (8) | -24.9 (8) | 0.004 | <0.001 |
| **Lung (N)** | -29.7 (4) | -53.7 (4) | -6.2 (11) | -15.9 (11) | 0.13 | 0.51 |
| **Liver (N)** | -13.8 (3) | -14.4 (3) | -23.8 (6) | -45.9 (6) | 0.71 | 0.90 |
| **Lymph Node (N)** | -41.5 (4) | -69.7 (4) | -12.1 (4) | -42.0 (5) | 0.34 | 0.55 |
| **Mean change (N)** | -35.4 (19) | -64.7 (20) | -12.7 (29) | -28.7(30) | 0.015 | 0.03 |

N = number of patients involved for computing the mean response.

^a^ p value corresponding to the test for a significant inter‑arm difference according to the location of the disease and each quantitative imaging biomarker (Wilcoxon-rank test).

Data are the mean stratified response (%) by location in each arm, considering either the diameter or volume of tumors and the weighted mean of stratified responses. For each patient, the stratified change of tumor burden was computed by adding up target tumor sizes from the same tumor location.

**Table I.2: Intra‑arm pair‑wise comparisons of proportional change in tumor diameter from baseline to Week 12 at different disease locations with removal of outliers**

|  | **Varlitinib + Capecitabine** | | **Lapatinib+ Capecitabine** | |
| --- | --- | --- | --- | --- |
|  | **Difference [CI] (%)** | **p value^a^** | **Difference [CI] (%)** | **p value^a^** |
| **Liver-breast** | 46.55 [8.84; 84.26] | 0.01 | -10.08 [-58.54; 38.38] | 0.93 |
| **Lung-breast** | 13.59 [-15.61; 42.80] | 0.55 | 13.08 [-29.48; 55.64] | 0.83 |
| **Lymph Node-breast** | 0.03 [-29.18; 29.24] | 1.0 | 1.55 [-53.39; 56.50] | 0.99 |
| **Lung-liver** | -32.95 [-74.27; 8.36] | 0.14 | 23.16 [-23.17; 69.50] | 0.52 |
| **Lymph node-liver** | -46.51 [-87.82; -5.21] | 0.02 | 11.64 [-46.28; 69.56] | 0.94 |
| **Lymph Node-lung** | -13.56 [-47.29; 20.17] | 0.66 | -11.52 [-64.61; 41.56] | 0.93 |

CI = Confidence interval.

^a^ p value of the test of a significant difference in responses (Test of Tukey Honest Significant Differences).

Data are the mean difference in response (%) for each given pair‑wise comparison with the corresponding 95% confidence interval.

**Table I.3: Intra-arm pair‑wise comparisons of proportional change in tumor volume from baseline to Week 12 at different disease locations with removal of outliers.**

|  | **Varlitinib + Capecitabine** | | **Lapatinib+ Capecitabine** | |
| --- | --- | --- | --- | --- |
|  | **Difference [CI] (%)** | **p value^a^** | **Difference [CI] (%)** | **p value^a^** |
| **Liver-breast** | 69.79 [0.17; 139.42] | 0.05 | -21.09 [-104.05; 61.85] | 0.89 |
| **Lung-breast** | 30.49 [-32.27; 93.25] | 0.52 | 8.35 [-66.28; 82.99] | 0.99 |
| **Lymph Node-breast** | 14.47[-48.29; 77.23] | 0.91 | -17.15 [-104.72; 70.41] | 0.95 |
| **Lung-liver** | -39.31 [-119.08; 40.46] | 0.51 | 29.45 [-51.50; 110.40] | 0.75 |
| **Lymph node-liver** | -55.32 [-135.09; 24.44] | 0.23 | 3.94 [-89.06; 96.95] | 0.99 |
| **Lymph Node-lung** | -16.01 [ -89.87; 57.83] | 0.92 | -25.51 [-111.18; 60.16] | 0.84 |

CI = Confidence interval

^a^ p value of the test of a significant difference in responses (Test of Tukey Honest Significant Differences).

Data are the mean difference in response (%) for each given pair wise comparison with the corresponding 95% confidence interval

**Linear system for independent tumors: balanced proportion of tumor location**

The proportion of tumor at each tumor location was balanced according to the averaged proportion of the two arms. Tests for a significant difference in the responses using tumor diameter and volume as QIBs revealed p=0.145 and p=0.006, respectively.

1. Tumor diameter

1/37 * (11* -50.19% + 11.5* -29.96% +8.5 * -8.64% +6*-46.15%) = -33.7% Equation. I.1

1/37 * (11* -16.15% + 11.5* -12.17% + 8.5 * -21.55% + 6*-31.86%) = -18.7% Equation. I.2

1. Tumor volume

1/37 * (11* -85.06% + 11.5* -56.31% + 8.5 * 7.23% +6*-71.7%) = -52.75% Equation. I.3

1/37 * (11* -30.90% + 11.5* 16.68% + 8.5 * -44.71% + 6*-46.48%) =-21.8% Equation. I.4

**Linear system for stratified tumor burden per patient: Balanced proportion of patient having tumors at each location.**

The proportion of patients having tumors at each tumor location was balanced in both treatment arms. Before balancing the data, stratification yielded 21 and 31 patients in the VC and LC arms, respectively. Testing for a significant difference in the responses using tumor diameter and volume as QIBs, we found p=0.26 and p=0.11, respectively.

1. Tumor diameter

1/26 * (8.5* -49.6% + 8* -29.7% + 5 * 0.4% + 4.5*-43.3%) = -32.8% Equation I.5

1/26 * (8.5* -13.7% + 8* -15.5% + 5 * -23.8% + 4.5*-29.7%) = -18.9% Equation I.6

1. Tumor volume

1/26 * (8.5* -84.2% + 8* -53.7% + 5 * 23.28% + 4.5*-69.73%) = -51.64% Equation I.7

1/26 * (8.5* -24.86% + 8* -17.24% + 5 * -45.96% + 4.5*-42.02%) =-29.54% Equation I.8
